# The Metabolic Score for Visceral Fat (METS-VF) as a predictor of diabetes mellitus: Evidence from the 2011–2018 NHANES study

**DOI:** 10.1371/journal.pone.0317913

**Published:** 2025-02-11

**Authors:** Harshita Tripathi, Amandeep Singh, Farheen, Bindu Prakash, Dharmendra Kumar Dubey, Prayas Sethi, Ranveer Singh Jadon, Piyush Ranjan, Naval K. Vikram

**Affiliations:** 1 Department of Medicine, All India Institute of Medical Sciences (AIIMS), New Delhi, India; 2 Department of Biostatistics, BK Autonomous State Medical College, Uttar Pradesh, India; The Chinese University of Hong Kong, HONG KONG

## Abstract

**Introduction:**

With 42.4% of obese US population, the prevalence of diabetes mellitus (DM) is increasing. Visceral fat, even at the same body mass index, has significant deleterious effects. This study aims to investigate the association between the Metabolic Score for Visceral Fat (METS-VF) and DM.

**Methods:**

The study utilized data from NHANES dataset, covering cycles from 2011 to 2018. Multivariate logistic regression and Receiver Operating Characteristic (ROC) analysis assessed the association between METS-VF and DM. Additionally, the study compared visceral adipose tissue (VAT) measurement indices to METS-VF for the prediction of DM.

**Results:**

In 3,445 participants, METS-VF was positively associated with diabetes [(OR 6.8; 95% CI 5.3–8.6) (AUC 0.791; 95% CI 0.768–0.814)], the association increased across quartiles (METS-VF >6.5; OR 53.8; 95% CI 0.17–169).METS-VF significantly identifies diabetes compared to other VAT indices (LAP, VAI, waist circumference, and WHtR). Additionally premenopausal females with BMI >25 and METS-VF >6.5 are at a higher risk of developing diabetes.

**Conclusion:**

METS-VF positively associates with the prevalence of diabetes. It is an effective score compared to surrogate markers for VAT measurement. Routine screening of VAT using METS-VF score could be implemented in daily clinical settings and large-scale epidemiological studies to help identify early diabetes.

## 1. Introduction

The prevalence of obesity in the US has been rapidly increasing and has reached up to 42.4% [1]. However, despite an 11.3% [2] prevalence of diabetes in the US population, only 30% of obese Americans develop diabetes mellitus (DM) [3], indicating that obesity is not the sole determinant of DM. Furthermore, there is increasing evidence that, at a given body mass index (BMI), the type of excess fat is an important predictor of metabolic diseases [4]. Compared to subcutaneous fat, visceral adipose tissue (VAT) is significantly associated with insulin resistance (IR) [5] because it releases proteins that contributes to low-grade chronic inflammation [6], adipokine dysregulation [7], and impairs glucose and lipid metabolism. This leads to the development of metabolic diseases such as diabetes mellitus, dyslipidemia, hypertension, cardiovascular events [8], and all-cause mortality [9]. Thus, the term "visceral adiposity" has frequently been used and has recently become a focus of research interest in DM.

Despite the significant metabolic burden and clinical relevance of evaluating visceral adiposity, its application in everyday clinical practice is limited by equipment and technical difficulties. Its assessment is challenging as anthropometric measures like waist circumference, waist-hip ratio, and waist-height ratio (WHtR) are unable to precisely measure it [10]. The gold standard for clinical assessment of visceral adiposity includes magnetic resonance imaging (MRI), computed tomography (CT), and dual-energy X-ray absorptiometry (DXA), but they are not suitable for large populations due to their expensive testing costs, exposure to harmful radiation, and complex procedures. Thus, indices that can provide accurate measurements of visceral obesity may be more helpful when assessing risk of diabetes mellitus.

The Metabolic Score for Visceral Fat (METS-VF) [11] is a novel scoring tool that includes the Metabolic Index of Insulin Resistance (METS-IR), waist-to-height ratio, age, and gender for the estimation of visceral fat and has been a good indicator for predicting diabetes [12, 13] and hypertension [14]. METS-VF has exhibited high agreement with the gold standard and showed superiority over CT [15], MRI [11], DXA, [16] and BIA [11]. It also has good risk assessment and predictive power for metabolic diseases closely related to visceral adiposities such as chronic kidney disease [17], hypertension, and hyperuricemia [18].

Despite the plethora of evidence, that visceral fat plays a fundamental role in the development of diabetes mellitus and METS-VF accurately quantifies visceral fat, the association between METS-VF and diabetes risk has only been explored in the Chinese population [11, 12]. Its predictive power for the risk of diabetes in the US population is currently unknown. Additionally, the previous study did not find any association between METS-VF and DXA-based visceral fat but only used VAP, LAI, WC, and WHtR as markers of visceral adiposity. Motivated by this gap, our study aims to undertake a cross-sectional analysis to assess the predictive ability of METS-VF for diabetes in the US population using data from the National Health and Nutrition Examination Survey (NHANES). At the same time, we also aim to compare METS-VF with other visceral adiposity indices like DXA-based visceral fat, WHtR, Lipid Accumulation Product Index (LAP), and Visceral Adiposity Index (VAI) for detecting the risk of diabetes.

## 2. Materials and methods

### 2.1 Data source and study population

**Fig 1:**
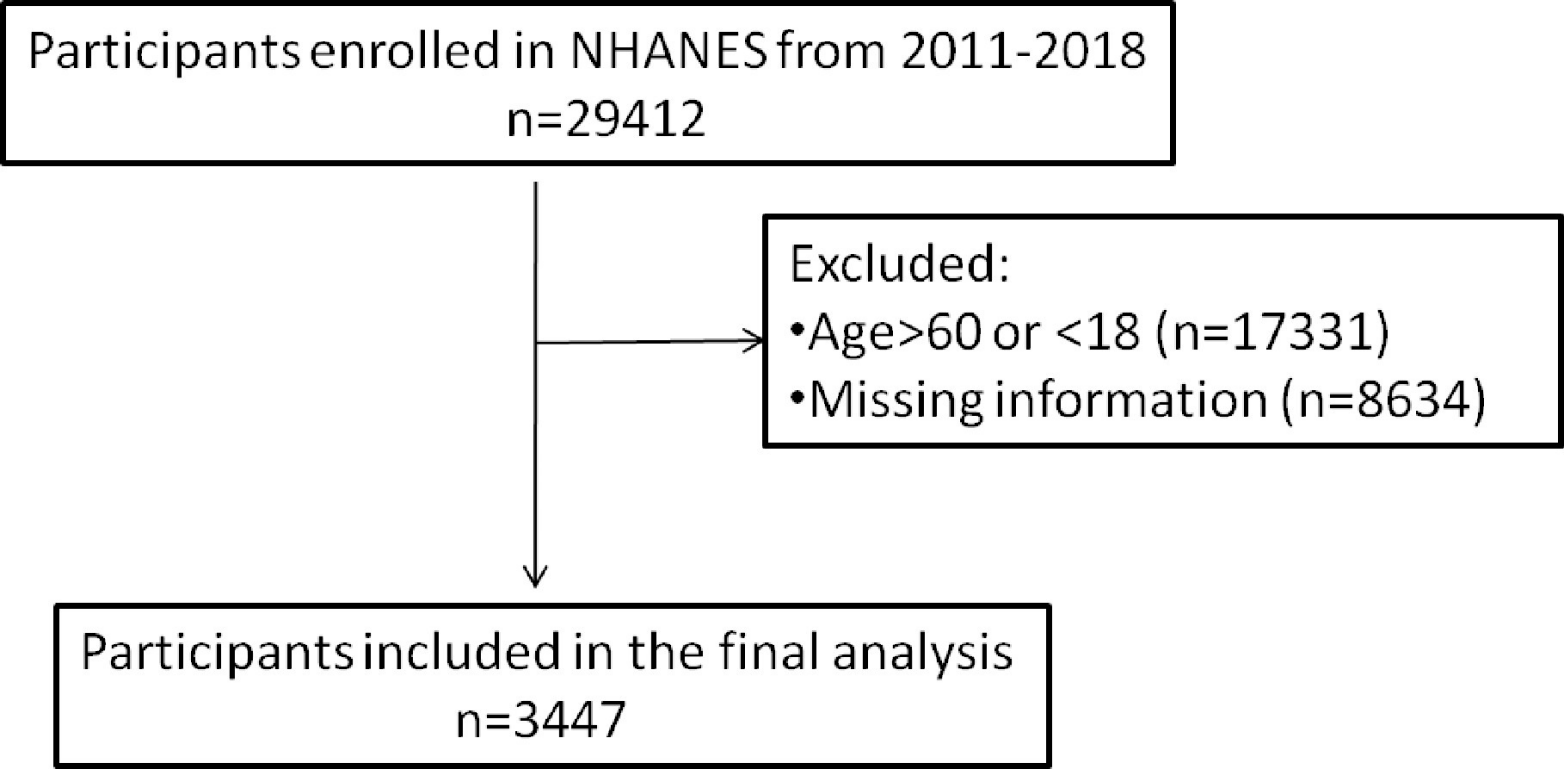
Flowchart for participants.

The NHANES database was used for this study. As part of the implementation of the NHANES protocols, the National Center for Health Statistics (NCHS) Research Ethics Review Board reviewed and standardized all procedures in accordance with the Human Research Subject Protection Policy of the US Department of Health and Human Services (HHS). A written informed consent was also obtained from all the participants or their parents or guardians. A total of four NHANES survey cycles (2011–2018) were selected for cross-sectional studies in this study. In the survey 29,412 people participated and only adults were included so minors aged under 18 and above 60 were excluded (n = 17,331). We also excluded participants with the missing information (fasting plasma glucose = 8,634). The final study included 3447 participants (Fig 1).

### 2.2 METS-VF and other covariates

The main outcome in this study included METS-VF score. Abdominal adiposity indicators included visceral adipose tissue (VAT) mass and subcutaneous adipose tissue (SAT) measured by whole-body DXA using Hologic Discovery A densitometers. Anthropometric adiposity indicators included BMI, waist circumference, and waist-to-height ratio (WHtR). METS-VF score was calculated by combining insulin resistance index (METS-IR), waist-to-height ratio (WHtR), age and sex using the formula.

METS VF = 4.466 + 0.011[(Ln (METS-IR))3]+ 3.329 [(Ln (WHtR))3]+0.319 (sex) + 0.594 (Ln (age)) [11] (where GLU is expressed in mmol/L, TG in mmol/L, BMI in kg/m^2^, HDL-C in mmol/L, age in years, and sex was a binary response variable (men = 1, women = 0, WHtR = WC(cm)/HT(cm), METS-IR = Ln ((2×GLU)+TG)×BMI)/ (Ln(HDL-C). Other adiposity indices included LAP and VAI which were calculated using the formula [13] as follows

VAI _men_ = (WC /39.68+1.88*BMI) * (TG/1.03) * (1.31/HDL-C)

VAI _women_ = (WC /36.58+1.89*BMI) * (TG/0.81) * (1.52/HDL-C)

LAP _men_ = (WC-65)*TG

LAP _women_ = (WC-58)*TG

Other covariates in this study obtained by in-person interview included age, gender (male/female), age (years), and survey cycle (2011–2012, 2013–2014, 2015–2016, and 2017–2018). Socio-economic status included education level (less than 10, high school and college) and income ($ 0–20,000, $20, 2000–55,000, $55,000–75,000, $75,000 to $99,999–100,000, and over $100,000. Self-reported lifestyle factors included alcohol consumption (yes or no), smoking status (smoking yes or no), and diabetes mellitus (yes or no). Cardiovascular risk factors included diabetes, triglyceride, total cholesterol, and high-density lipoprotein. Dietary intake factors included energy intake, protein intake, fat intake, and sugar intake.

### 2.3 Data collection and definitions

Diabetes was defined as fasting plasma glucose (FPG) ≥7·0 mmol/land/or use of insulin oral hypo-glycaemic medication [19].

Uncontrolled diabetes was defined as FPG>8.5 mmol/l [20, 21].

### 2.4 Statistical methods

A multistage sampling design employed in selecting a representative non-institutionalized US population was illustrated through the application of NHANES sampling weights, stratifications, and clustering in all statistical analyses. Weights provided by the dataset, the “examination sample weights” were used, and since the 4-year cycle was used the 2-year sample weights were divided by four. Categorical variables were presented as weighted frequency (%). Continuous variables were presented as weighted survey means (SD) or median (interquartile range) as appropriate. The logistic regression model along with the hazard ratios and 95% CI and Receiver operating characteristics (ROC) were used to associate diabetes with the metabolic score and the four models of METS-VF. Model 1 included unadjusted covariates. Age, gender, and education level were adjusted in Model 2. Alcohol intake, smoking, and fat intake were adjusted in Model 3. Other variables like BMI, WHtR, WC, LAP, VAI, DXA based visceral fat area were adjusted in Model 4. Multivariate logistic regression models and ROC curves were used to compare METS-VF score and other surrogate markers of visceral fat for the association with diabetes. Further, a sub-group analysis was also performed stratifying by sex (men and women), age (<45 and ≥45), BMI (<25and ≥25), and controlled vs. uncontrolled glucose values. A p< 0.05 was considered significant. SPSS version 29 was used to conduct the analyses.

## 3. Results

### 3.1 Participant characteristic

Our study included 3,442 participants aged 18 to 59 years, of which 46.3% were male. Compared to non-diabetic participants, diabetic patients were older, had lower educational attainment, and were less likely to have higher income status. They also had higher consumption of alcohol, smoking habits, and a higher intake of fat. Baseline characteristics of the participants are shown in Table 1.

**Table 1:** Baseline characteristics of the study participants.

Characteristics	Total (n = 3447)	Diabetic (383)	Non-Diabetic (3064)	P value
	N (%)			
Male	1657 (46.3)	217 (56.6)	1440 (46.9)	0.001
Female	1790 (51.9)	166 (43.3)	1624 (53)	
Education				
Less than 10^th^ grade	644 (18.6)	102 (26.6)	542 (17.6)	
High School	669 (18.7)	79 (20.6)	590 (19.2)	0.001
College	1872(88.5)	200 (52.2)	1672 (54.5)	
Income				
0–20,000 ($)	687 (19)	75 (19.5)	612(19.9)	
20,2000–55,000 ($)	1186 (35)	149 (38.9)	1037 (33.8)	
55,000–75,000 ($)	349 (10)	54 (14)	298 (9.7)	0.165
75,000 to 99,999 ($)	343 (9.9)	36 (9.39)	307 (10.01)	
100,000 ($) and Over	610 (17.6)	46 (12)	564 (18.4)	
Alcohol Intake	412 (11.3)	72 (18.7)	340 (11)	0.001
Smoking	776 (22)	94 (24.5)	682 (22.2)	0.001
	**Median (Interquartile range)**			
Age (years)	38.2 (22)	50 (14)	37 (21)	0.001
Energy (kcal)	2000.3 (1159)	2023.5 (1416)	1995 (1141)	0.675
Protein (g)	76.6 (49.2)	76.5 (49.1)	76.6 (49.1)	0.633
Fat (g)	78.3 (52.2)	81.3 (59.4)	78.3(51.4)	0.089
Sugar (g)	92.5 (84)	82.1 (90.8)	94 (83.6)	0.194
BMI(kg/m2)	28.3 (9.5)	31.7 (8.9)	27.7 (9)	0.001
WC (cm)	96.3 (23.2)	10.9.0 (23.3)	94.7 (22.9)	0.001
WHtr	0.57 (0.13)	0.64 (0.13)	0.56 (0.13)	0.001
LAP	362.5 (285.3)	515.5 (291.8)	343.3 (265.9)	0.001
VAI	3.1 (4.08)	5.1 (5.8)	2.7(3.65)	0.001
METS-VF	6.2 (0.83)	6.6 (0.44)	6.1 (0.85)	0.001
TC (mmol/L)	10.26(2.9)	11.1 (3.7)	10.2 (2.8)	0.001
TG (mmol/L)	4.8 (4.3)	7 (5.8)	4.5 (3.9)	0.001
HDL (mmol/L)	2.83 (1.1)	2.49 (0.93)	2.88 (1.1)	0.001
Fasting Glucose (mmol/L)	5.5 (0.83)	8.1 (4)	5.4 (0.61)	0.001
Subcutaneous Fat Area (cm^2^)	325.5 (254.3)	393.3(261.2)	312.4 (240.9)	0.001
Visceral Fat Area (cm^2^)	96.7 (82.1)	157.2 (88.9)	90.2 (77.5)	0.001

Data presented as mean ±SD, median (min-max) or n (%) as appropriate. Significant at p<0.05.

BMI: Body Mass Index; WC: Waist Circumference; WHtR: Waist-to- height ratio; LAP: Lipid Accumulation Product Index; VAI: Visceral Adiposity Index; METS-VF: Metabolic Score for Visceral Fat; TC: Total cholesterol; TG: Triglycerides; HDL: High Density Lipoprotein.

### 3.2 Association of METS-VF with the prevalence of diabetes

There was a significant association of the high METS-VF score with the prevalence of diabetes (OR 6.8; 95% CI- 5.68–8.3; p-0.001) and this association remained significant even after adjusting for the potential confounding factors in different models (Table 2).

**Table 2:** Multivariate logistic regression analysis for the association between METS-VF score and diabetes prevalence.

Models	Odd’s Ratio (95% CI)	P value
Model 1	6.8 (5.68–8.3)	0.001
Model 2	7.23 (5.00–10.46)	0.001
Model 3	9.69 (5.70–16.45)	0.001
Model 4	12.9 (5.1–32.3)	0.001

Data presented as OR (95%CI). Significant at p<0.05.

Model 1: Unadjusted

Model 2: Adjusted for Age, gender, income

Model 3: Adjusted for smoking, alcohol intake and fat intake

Model 4: Adjusted for BMI, WC, WHtR, LAP, VAI, DXA-based visceral fat

BMI: Body Mass Index; WC: Waist Circumference; WHtR: Waist-to- height ratio; LAP: Lipid Accumulation Product Index; VAI: Visceral Adiposity Index; DXA: Dual-energy X-ray absorptiometry; METS-VF: Metabolic Score for Visceral Fat

**Fig 2:**
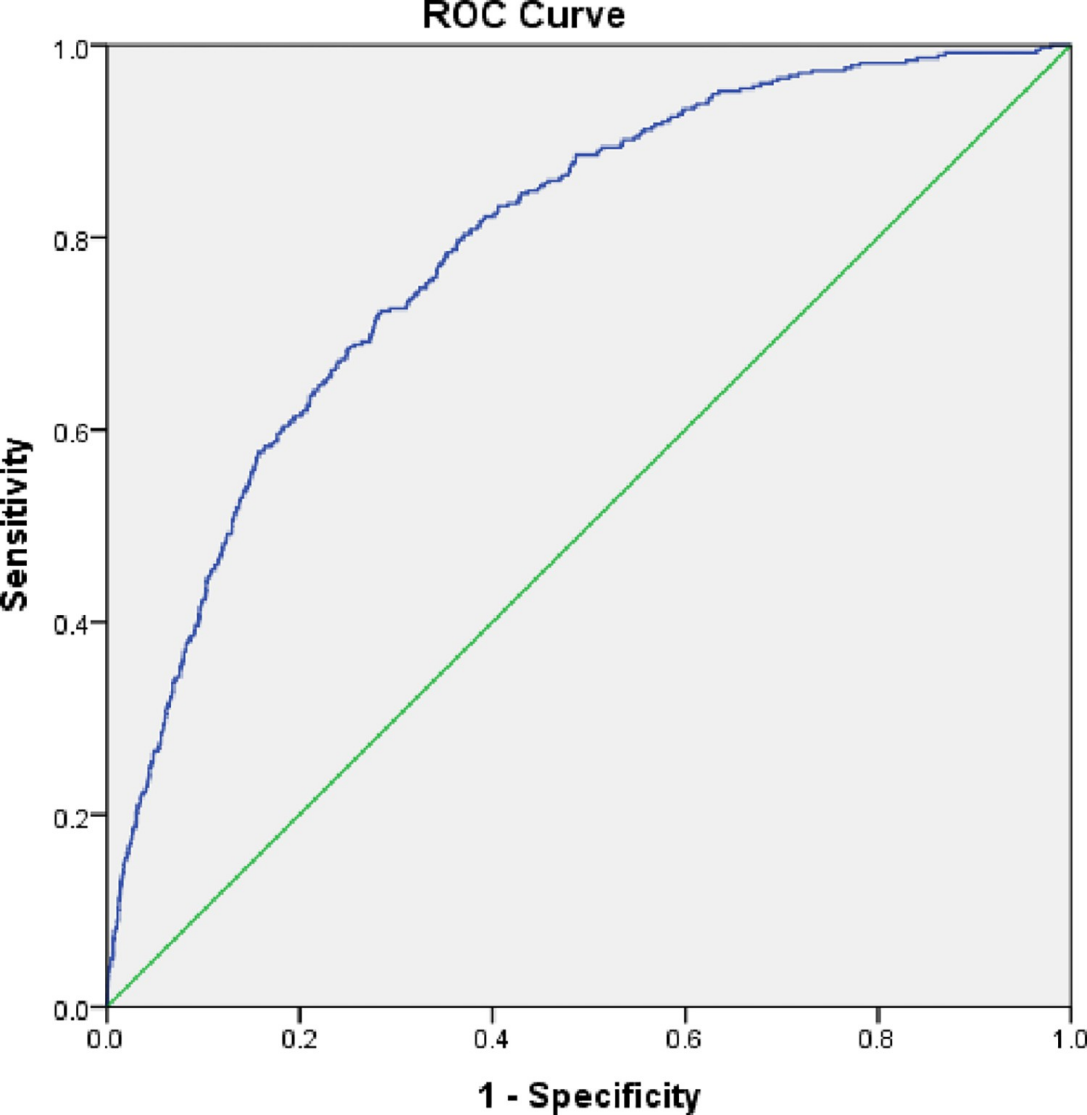
ROC analysis to test the accuracy of METS-VF for diabetes prevalence.

The ROC analysis confirmed the above results as METS-VF had a high area under curve of 0.791 (95% CI- 0.768–0.814; p-0.001) for the detection of diabetes with a high Youden index of 0.428 (Fig 2, Table 3).

**Table 3:** Areas under the receiver operating characteristic curves for METS-VF and diabetes mellitus risk.

Variable	AUC	95% CI	Cut-off	P value	Sensitivity	Specificity	Youden Index
METS-VF	0.791	0.768–0.814	6.38	<0.001	72.6	29.8	0.428

Significant at p<0.05

METS-VF: Metabolic Score for Visceral Fat; AUC: Area Under the Curve

The cut-off of METS-VF for the detection of diabetes was 6.38 for the current data. Furthermore, when METS-VF was grouped by quartiles, we found that the positive association became more pronounced with the increasing quartiles of METS-VF and a score of >6.5 had the highest association with occurrence of diabetes (P value<0.01) (Table 4).

**Table 4:** Multivariate logistic regression analysis for the association of quartiles of METS-VF and prevalence of diabetes mellitus.

	Quartile 1			Quartile 2			Quartile 3			Quartile 4		
**Range**	<5.6			5.6–6.16			6.17–6.5			>6.5		
**No. of cases**	823			882			1122			620		
	OR	95% CI	P value	HR	95% CI	P value	HR	95% CI	P value	HR	95% CI	P value
**Model 1**	1.13	0.30–4.23	0.85	13.03	9.2–18.3	0.001	13.03	9.25–18.3	0.001	53.8	0.17–169	0.001
**Model 2**	0.87	0.22–3.38	0.84	7.34	5.08–10.6	0.001	7.34	5.08–10.6	0.001	0.69	0.51–0.95	0.001
**Model 3**	0.67	0.069–6.51	0.73	9.29	5.5–15.6	0.001	9.23	5.5–15.6	0.001	27	4.84–150	0.001
**Model 4**	10	5.6–12.3	0.016	12.9	5.1–32.3	0.001	12.92	5.1–32.3	0.001	58.97	4.9–701.1	0.001

Model 1: Unadjusted

Model 2: Adjusted for age, gender education

Model 3: Adjusted for alcohol intake, smoking, fat intake

Model 4: Adjusted for BMI, WHtr, WC, LAP, VAI, Visceral fat area on DXA

Significant at p<0.05

BMI: Body Mass Index; WC: Waist Circumference; WHtR: Waist-to- height ratio; LAP: Lipid Accumulation Product Index; VAI: Visceral Adiposity Index; DXA: Dual-energy X-ray absorptiometry; METS-VF: Metabolic Score for Visceral Fat

### 3.3 Comparison of association of surrogate visceral fat indices and METS-VF with prevalence of diabetes

**Fig 3:**
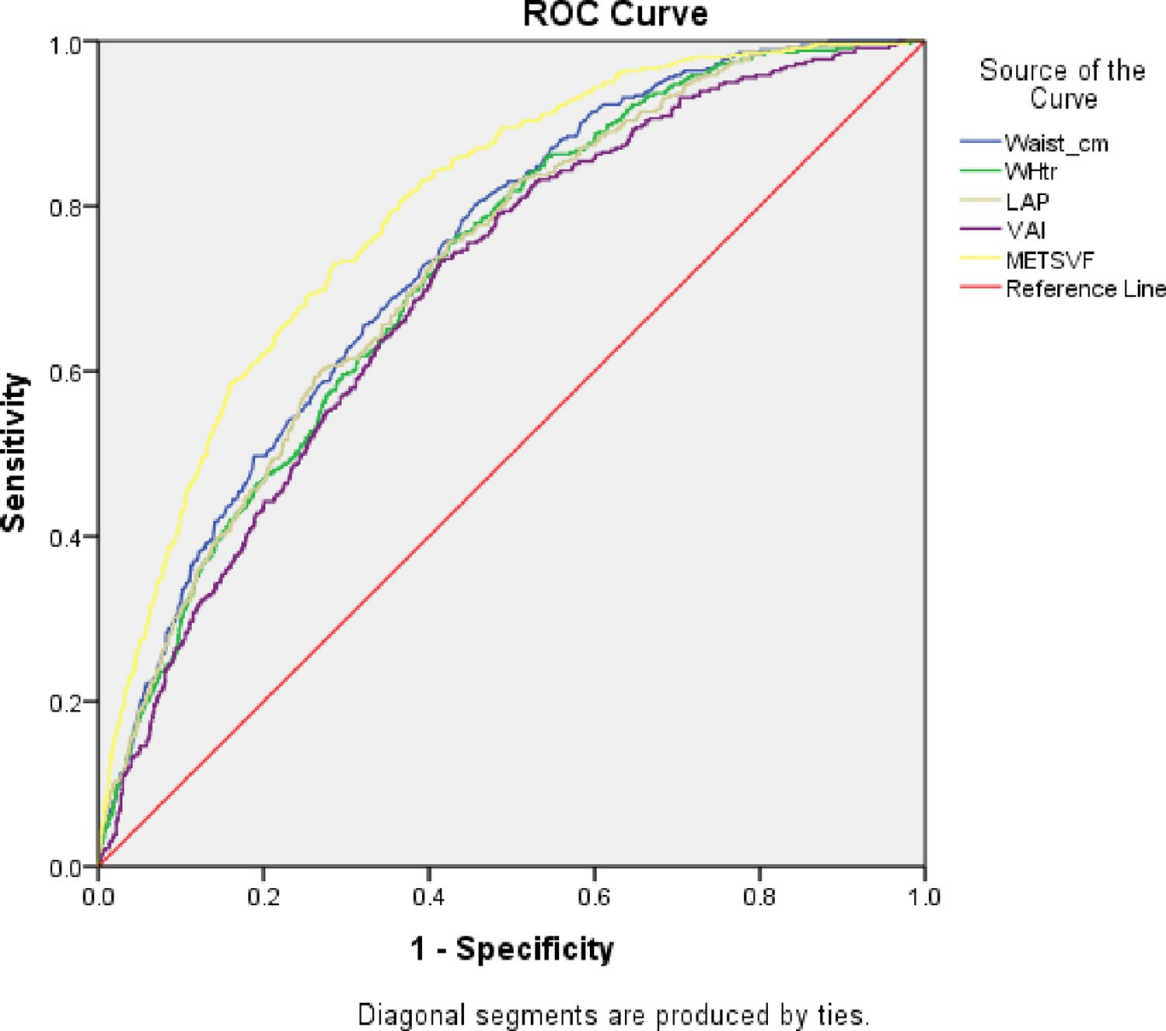
ROC analysis to test the accuracy of METS-VF to predict diabetes in comparison to other visceral fat surrogates.

METS-VF had the largest AUC for predicting diabetes (0.798; 95% CI-0.775–0.820; p = 0.001), significantly different from WC, LAP, VAI and WHtR (P<0·001) and the Youden index for METS-VF was the highest for all participants (Fig 3, Table 5).

**Table 5:** ComparisonofareasunderthereceiveroperatingcharacteristiccurvesforMETS-VF, LAP, VAI, WHtR, WC and BMI with diabetes mellitus risk.

Variable	AUC	95% CI	Cut-off	P value	Overall P value	Sensitivity	Specificity	Youden Index
METS-VF	0.798	0.775–0.820	6.38	<0.001	<0.001	72.6	29.8	0.42
LAP	0.727	0.701–0.752	329.6	<0.001		82.2	50.6	0.31
VAI	0.702	0.676–0.729	4.17	<0.001		62.9	33.8	0.29
WHtR	0.723	0.698–0.748	0.58	<0.001		72.4	41.4	0.31
WC	0.740	0.715–0.764	94.9	<0.001		80.9	47.7	0.33

Significant at p<0.05

AUC: Area Under the Curve; BMI: Body Mass Index; WC: Waist Circumference; WHtR: Waist-to- height ratio; LAP: Lipid Accumulation Product Index; VAI: Visceral Adiposity Index; DXA: Dual-energy X-ray absorptiometry; METS-VF: Metabolic Score for Visceral Fat

### 3.4 Subgroup analysis

The subgroup analysis showed that at a higher quartile of METS-VF, females (OR 148.4 vs. 72.3, 95% CI- 23–956; p-0.001), at a premenopausal age (age<45) (OR 72.3 vs. 36.9; 95% CI 4.59–1139; p-0.002) and those with BMI>25 (OR 54.5 vs. 0; 95% CI 17.3–171.2; p-0.001) were significantly associated with an increased risk of diabetes (Table 6).

**Table 6:** Multivariate logistic regression analysis for the association of quartiles of METS-VF and prevalence of diabetes mellitus.

	Quartile 1			Quartile 2			Quartile 3			Quartile 4		
Range	<5.6			5.6–6.16			6.17–6.5			>6.5		
**No. of cases**	823			882			1122			620		
	OR	95% CI	P value	OR	95% CI	P value	OR	95% CI	P value	OR	95% CI	P value
**Male**	0.869	0.23–3.1	0.833	8.5	5.6–12.7	0.001	8.5	5.6–12.7	0.001	25.5	5.8–108.3	0.001
**Female**	9.08	6.6–15.6	0.22	27	14.7–50	0.001	27.16	14.7–50.1	0.001	148.4	23–956	0.001
**Age <45**	1.003	0.263–3.826	0.996	8.82	5.31–14.65	0.001	8.82	5.3–14.6	0.001	72.3	4.59–1139	0.002
**Age >45**	9.640	0.01–1.98	0.449	8.7	5.3–14.5	0.001	8.7	5.3–14.5	0.001	36.9	9.5–143.8	0.001
**BMI <25**	1.16	0.29–4.63	0.828	6.99	3.34–14.64	0.001	6.9	3.34–14.6	0.001	0.00	0.00	0.00
**BMI ≥25**	21.2	0.01–126	0.701	26.06	16.08–42.23	0.001	26.06	16.08–42.23	0.001	54.4	17.3–171.2	0.001

Significant at p<0.05

BMI: Body Mass Index

We also found that participants with uncontrolled blood glucose had high METS-VF (OR 0.06; 95% CI: 0.87–1.1) (Table 7).

**Table 7:** Logistic regression for the association of METS-VF with controlled diabetes vs. uncontrolled diabetes.

Variable	Controlled diabetes (n = 357)	Un-controlled diabetes (n = 163)	P value
METS-VF	5.5±1.1	6.57±0.34	0.001
OR (95% CI)	0.94 (0.82–1.1)	0.06 (0.87–1.1)	

Significant at p<0.05. METS-VF: Metabolic Score for Visceral Fat

We may conclude that overweight female participants at a premenopausal age having METS-VF score of more than 6.5 are at high risk of developing diabetes.

### 3.5 Association of METS-VF in participants with controlled diabetes vs. uncontrolled diabetes

Participant having diabetes with uncontrolled sugar had significantly higher METS-VF (6.57±.34 vs. 5.5±1.1 [(OR- 0.06;95% CI: 0.87–1.1) vs. (0.94; 95%CI: 0.82–1.1); p-0.001] (Table 7).

## 4. Discussion

In this nationally representative sample and cross-sectional study, we tried to explore the association between METS-VF and prevalence of diabetes mellitus among US adults. The results demonstrated a significant association between METS-VF score and diabetes prevalence, which remained significant even when adjusted for the confounding variables and increased across the quartiles of METS-VF. The sub-group analyses also confirmed this positive association of METS-VF with the diabetes, when stratified on the basis of age, gender and BMI. Moreover, amongst the known surrogate indices of visceral adiposity (WC, WHtR, LAP, VAI),METS-VF had the highest AUC and Youden index for the diabetes prevalence, which was statistically significant (p<0.001).

Studies have proven that as compared to the subcutaneous fat, visceral fat accumulation has far more deleterious effects and remains an independent risk factor for diabetes incidence [22]. A recent study result proved that visceral fat is an independent predictor of diabetes mellitus (OR 1·60; 95% CI 1·10, 2·30) [23]. In other similar studies by Wu et al., [24] Liu et al., [13] Chen et al., [25] and Neeland et al., [7] visceral adiposity remains independently associated with diabetes. The gold standard to measure visceral adiposity in the clinical setting includes MRI, CT and DXA. However, the above used modalities have certain drawbacks as they are expensive, require special equipment, expose the participants to radiation and need specialist interpretation. Therefore, in the current study we used a novel tool for the estimation of VAT i.e., METS-VF which utilizes the parameters which remain common clinically available, including fasting glucose, triglyceride, insulin resistance, age, gender, and WHtR [8] with diabetes. Our results found a strong and significant association between METS-VF and the prevalence of diabetes as compared to other VAT measuring indices including DXA-based visceral fat mass. A study by Feng et al., [11] showed similar results that risk of diabetes strongly associated with METS-VF. Recently, Yang et al., [12] also showed that METS-VF is a significant predictor for the future occurrence of diabetes. Both these studies included Chinese population and did not include DXA-based VAT measurement. In our prospective study which was representative of the US population, we also found a significant association of METS-VF with the diabetes mellitus prevalence (OR 6.8; 95% CI 5.68–8.3; p-0.001). Additionally, on multivariate logistic regression analysis, when the possible covariates were adjusted, METS-VF remained significantly associated with diabetes mellitus (OR 6.8; 95% CI 5.3–8.6; p-0.001). METS-VF demonstrated high AUC (0.791; 95% CI- 0.768–0.814;p-0.001) and a high Youden Index (0.42) confirming this strong association. Our study also illustrated that prevalence of diabetes (OR: 53.8: 95% CI-0.17–169;p-0.001) was significantly higher in the participants falling in the highest quartile of METS-VF, which was similar to the result by Feng et al [11].

The routinely used anthropometric modality of VAT includes WC, BMI and WHtR. While, there is known association between these anthropometric measurements and cardio-metabolic diseases, but their capability to differentiate between the subcutaneous and visceral fat is limited. Study results of Yang et al in 2023 [12] and Feng et al., in 2022 [11] demonstrated that METS-VF had the highest predictive accuracy for future diabetes (AUC value 0.77) compared to other indicators. Our study also evaluated the effectiveness of METS-VF compared to the laboratory-based estimators of VAT (LAP, VAI) and commonly used anthropometric measurements (WHtR, WC, BMI) in detecting the prevalence of diabetes mellitus in the current population. Our data showed a significantly higher AUC for METS-VF as compared to all the other VAT measuring indices for diabetes occurrence (OR-0.79; 95%CI- 0.775–0.820; p-0.001).

The exact underlying mechanism of VAT influencing the occurrence of diabetes mellitus remains unknown, however several plausible mechanisms have been proposed. First, VAT acts as a metabolically active endocrine organ, which accelerates the release of fat and decreases the re-esterification rate causing release of pro-inflammatory cytokines leading to insulin resistance [26]. Increased insulin level hinders lipolysis activity, which increases the free fatty acids (FFAs) concentration in the bloodstream. These FFAs accumulates in the muscle fibers which when broken down, convert to Diaclyglecerols (DAGs) and long chain acyl-CoAs contributing to insulin resistance [27]. The second mechanism includes the amplification of the rate of hepatic gluconeogenesis due to excess mesenteric triacylglycerol in the liver, leading to hepatic insulin resistance. The third mechanism involves an increased rate of lipid oxidation via FFAs, leading to acetyl CoA buildup, which is a rate-limiting step for gluconeogenesis, ultimately resulting in insulin resistance [28].

Our subgroup analysis showed that, the highest quartile of METS-VF(>6.5)significantly consists of pre-menopausal females (OR 72.3 vs. 36.9; 95% CI: 4.59–1139 vs. 9.5–143.8) with a higher BMI (OR 54.4 vs. 0; 95% CI: 17.3–171.2 vs. 0) and have a higher risk of developing diabetes. Similar to ours, a study has shown that young premenopausal women had significant VAT deposition irrespective of race [29]. Few other studies also have shown age related increase of fat mass, preferentially visceral fat mass in females [30]. Further, the participants with diabetes who had uncontrolled sugar had higher visceral fat accumulation as estimated by METS-VF [6.57±0.34 vs. 5.5±1.1 (OR 0.06 vs. 0.94; 95% CI: 0.87–1.1 vs. 0.82–1.1)] which may be due to the reason that abnormal amount of glucose in the bloodstream results in fat accumulation and disturbance in the lipid metabolism [31].

The strengths of this present study include usage of NHANES dataset which includes a large, nationally representative sample of non-institutionalized US civilians and follows standardized protocols for examination and data collection. Further, this is the first study to investigate relationship of diabetes and METS-VF score in the US population, which accurately quantifies visceral fat. Additionally this study compared various VAT measurement indices (METS-VF, VAI, LAP, WHtR, and WC) along with DXA measured visceral fat, which was not addressed in previous studies. Few limitations of the study to be noted include, as this was a cross-sectional study, follow-up data was not available and therefore, cause and association of developing diabetes and METS-VF could not be observed. Finally, as this is an observational study, even after adjusting for covariates, other confounding factors may affect our results.

## 5. Conclusion

Our results demonstrate a strong association of METS-VF score with the prevalence of diabetes in the US adults regardless of gender, age, alcohol intake or smoking habit. METS-VF proved to be an effective tool for VAT measurement compared to other convenient surrogate markers. Therefore, METS-VF could be used in daily clinical setting and in large-scale epidemiological studies to identify the risk of diabetes.
